# Supplementary material for: Bile Acids: Major Regulator of the Gut Microbiome
Source: Microorganisms. 2022 Sep 6;10(9):1792. doi: 10.3390/microorganisms10091792 (PMC9502002; doi:10.3390/microorganisms10091792)
Supplement: Supplementary file 1 [file microorganisms-10-01792-s001.zip › microorganisms-1865360-supplementary.pdf]

Supplementary materials

Supplementary Table S1. Comparison between groups of relative quantity of microbiotas in colon and cecum.

a. Colon							
Relative quantity	Groups						Significance value*
	vehicle	UDCA	CA	CDCA	DCA	LCA	
Universal T†	22.90±18.07 a	22.94±9.36 a	23.75±11.35 a	18.80±8.56 a	60.25±57.84 a	42.78±48.04 a	NS (P= .596)
Bacteriotetes T†	16.35±8.71 a	49.13±63.58 a	33.02±4.81 a	15.16±2.16 a	13.28±3.76 a	40.65±21.56 a	NS (P= .515)
Firmicutes T†	5.78± 2.47 a	11.33±14.44 a	6.32±0.88 a	4.42±0.84 a	4.66±1.04 a	25.42±35.34 a	NS (P= .564)
b. Cecum							
Relative quantity	Groups						Significance value*
	vehicle	UDCA	CA	CDCA	DCA	LCA	
Universal T†	8.21±1.73 a	39.93±7.27 c	8.94±1.99 a	21.45±2.31 b	15.47±0.16 a,b	9.01±0.74 a	P= .000
Bacteriotetes T†	5.26±1.07 a	28.38±12.71 b	4.80±1.53 a	10.58±1.47 a	7.91±1.01 a	6.21±1.14 a	P= .001
Firmicutes T†	5.08±1.22 a	29.98±7.83 c	6.57±1.27 a,b	15.99±4.13 b	10.75±0.93 a,b	5.80±0.31 a	P= .000

* Statistical significances were tested by oneway analysis of variances among group.

† The same letters indicate non-significant difference between groups based on Tukey′s multiple comparison test.

Supplementary Table S2. Comparison of Firmicutes/Bacteroidetes ratio (F/B ratio) between colon and cecum.

Relative quantity of microbiota		Groups					
		vehicle	UDCA	CA	CDCA	DCA	LCA
Universal	Colon	22.90±18.07	22.94±9.36	23.74±11.35	18.80±8.56	60.25±57.84	42.78±48.04
	Cecum	8.21±1.73	39.93±7.27	8.94±1.99	21.45±2.31	15.47±0.16	9.00±0.74
Bacteriotetes	Colon	16.35±8.71	49.13±63.58	33.02±4.81*	15.16±2.16*	13.28±3.76	40.65±21.56
	Cecum	5.26±1.07	28.38±12.71	4.80±1.53*	10.58±1.47*	7.90±1.01	6.21±1.14
Firmicutes	Colon	5.78±2.47	11.32±14.44	6.32±0.88	4.42±0.84*	4.66±1.04*	25.42±35.34
	Cecum	5.08±1.22	29.98±7.83	6.57±1.27	15.99±4.13*	10.75±0.93*	5.80±0.31
F/B ratio	Colon	3.17±1.73	4.25±0.17*	5.37±1.53*	3.45±0.17*	2.86±0.55*	4.26±2.95
	Cecum	1.11±0.49	0.91±0.19*	0.73±0.17*	0.68±0.19*	0.73±0.03*	1.08±0.23

* The mean difference is significant at the 0.05 level (p< 0.05; t-test) between colon and cecum.
